# Tridimensional assessment of the mandibular angle in patients with different skeletal patterns by cone-beam computed tomography scans: a retrospective study

**DOI:** 10.1186/s12903-023-03074-z

**Published:** 2023-06-04

**Authors:** Murilo Miranda-Viana, Gabriel Mosso Moreira, Larissa Moreira de Souza, Yuri Nejaim, Francisco Haiter-Neto, Deborah Queiroz Freitas

**Affiliations:** 1 Department of Oral Diagnosis, Oral Radiology Area, Piracicaba Dental School, University of Campinas, Piracicaba, São Paulo, SP 13414-903 Brazil; 2 Oral Radiology Area, Dental School, Federal University of Mato Grosso do Sul, Campo Grande, MS Brazil

**Keywords:** Anatomy, Mandible, Cone-beam computed tomography

## Abstract

**Background:**

Since the muscles of chewing are involved in the region of the mandibular angle, important structures in surgical and orthodontic procedures, to study its morphological aspects and the possible influence of different patterns of skeletal development would be of interest. Thus, this study aimed to assess the influence of patient characteristics - such as sex, skeletal malocclusion (Class I, Class II, and Class III) and facial type (brachycephalic, mesocephalic, and dolichocephalic) - on the width, height, thickness, and volume of the mandibular angle, using cone-beam computed tomography (CBCT) scans.

**Methods:**

CBCT scans were assessed − 144 men and 154 women, total of 298 - and classified according to skeletal patterns (skeletal malocclusions and facial types). Width, height, and thickness of the mandibular angle were measured using OnDemand 3D software. The volumetric measures of the mandibular angle were obtained using the ITK-SNAP software. Analysis of Variance (multiway ANOVA) with Tukey’s post-hoc test compared the data, with a 5% significance level.

**Results:**

Among the factors studied, sex significantly influenced all the analyzed variables (height, width, thickness, and volume of the mandibular angle) (p < 0.05); in general, male individuals presented higher values than females. In some cases, the skeletal malocclusion and facial type factors influenced only the width and height variables (p < 0.05); in general, the Class III and dolichocephalic individuals presented higher values in relation to the other types of skeletal malocclusions and facial types.

**Conclusions:**

Variations in the craniofacial growth pattern, considering the different skeletal malocclusions and facial types, had some influence in the width and height dimensions of the mandibular angle. Furthermore, sex influenced all the studied variables.

## Introduction

In the craniomaxillofacial complex, muscles and bones are anatomically and functionally linked to allow movements. According to Wolff’s law, bone architecture and morphology depend on the load applied by the muscles [1] and, when mechanically stimulated, there is an increase in osteoclastic activity in the pressed area and in osteoblastic activity in the contralateral area [2]. Thus, the tension imposed on bones by muscles can generate changes in their size, shape and/or density [3].

Masseters and medial pterygoids muscles, which are the main elevators of the mandible, act together around the angle of the mandible to elevate the mandible and close the mouth, in addition to promoting the protrusion of the mandible. During these movements, the tension exerted by the muscles may differently influence the growth and morphology of the mandibular angle, and studies have found that prominent mandibular angles are often associated with hypertrophy of the masseter and medial pterygoid muscles [4]. On the other hand, mandibular angle resection, a surgical procedure to reduce the lower facial width, induces atrophy of the masseter muscle and reduction of its volume [5]. This corroborates the information in the literature that there are associations between the morphologies of these muscles and the bone structure of the mandibular angle.

Due to variations from normality in the individual’s growth pattern, bone structures can suffer adaptations and modify their morphology. Based on these aspects, several studies have used imaging examinations such as cone-beam computed tomography (CBCT) to understand these morphological changes 3, 6–9]. Previous studies have assessed, by means of CBCT scans, mandibular bone structures such as the mandibular head and mandibular angle [3, 7, 10–14]. However, the studies used non-proportional sample sizes with respect to sex and skeletal pattern of development and/or patients with a specific pathological disease, restricting the group under study. Moreover, many of these studies assessed only linear measures, without volumetric analysis, and established conclusions by the distance between mandibular head and mandibular angle, without individually analyzing the mandibular angle.

Considering that the mandibular angle region is influenced by the insertion of the masseter and medial pterygoid muscles, and that variations in the individual’s craniofacial morphology can alter the activities of these muscles, the authors hypothesized that, consequently, the bone characteristics of the mandibular angle could be modified. Therefore, the aim of the present study was to assess the influence of patient characteristics, such as sex, skeletal malocclusion, and facial type, on the height, width, thickness, and volume of the mandibular angle, using CBCT scans.

## Materials and methods

### Study design

This is an observational, cross-sectional, and retrospective study, which was initiated after approval by the local institutional review board (IRB) (protocol **#5.452.688)**.

### Sample selection

Initially, 340 CBCT scans from the database of a dental radiology clinic, acquired between the periods of January 2014 to December 2016 using an i-CAT® Next Generation device (Imaging Sciences International, Hatfield, PA) were selected for application of the eligibility criteria described below. The acquisition parameters used were in accordance with the manufacturer: 120 kVp (kilovoltage), 5 mA (milliamperage), 17.3 s (seconds) of scanning time, extensive field of view (FOV) − 23 × 17 cm (centimeters) and 0.3 mm voxel.

The inclusion criteria were patients of both sexes, aged 18 years or older, and who had never undergone orthognathic surgery. CBCT scans of patients with previous trauma and/or pathological lesions, syndromes, and presence of artifacts that could impair the assessment of the anatomical structures of interest were not included in the sample.

After applying the eligibility criteria, the final sample was 298 CBCT scans − 144 male individuals (18 to 64 years old, mean age 32.04 ± 12.48) and 154 female individuals (18 to 76 years old, mean age 30.87 ± 11.47). The scans were anonymized before data collection.

### Sample classification

Initially, each patient was classified according to the sex and skeletal pattern of development: skeletal malocclusion (Class I, II, and III) and facial type (brachycephalic, mesocephalic and dolichocephalic), which were the independent variables of the study. For this purpose, two previously trained examiners, with three years of experience in analysis of CBCT scans, assessed the scans, in consensus, using the Carestream Dental 3D Imaging software (version 3.10.9.0, Atlanta, Georgia, USA).

Skeletal malocclusions were established based on Steiner’s cephalometric norms for the SNA angle (angle determined by the cephalometric points: S (sella); N (nasion) and A (maxilla - subspinatus); SNB angle (angle determined by the cephalometric points: S (sella); N (nasion) and B (mandible - supramental); and ANB angle (angle determined by the cephalometric points: A (maxilla - subspinatus); N (nasion) and B (mandible - supramental) [15, 16]. The ANB angle value was determined by subtracting the SNA and SNB angles (ANB = SNA-SNB). If the ANB angle value was between 0 and 4, skeletal malocclusion represented Class I; ANB value greater than 4 represented Class II; and ANB value less than 0 (negative) represented Class III.

Regarding facial types, patients were classified according to the Vert index, [17] which corresponds to the arithmetic mean of five cephalometric measures: facial depth (Po-Or / N-Pog); facial axis angle (N-Ba / Pt-Gn); mandibular arch (Dc-Xi / Xi-Pm); lower facial height (Xi-ENA / Xi-Pm); and mandibular plane angle (Go-Me / Po-Or). Resulting values greater than 0.5 was classified as brachycephalic type; values less than − 0.5 was classified as dolichocephalic type; and values between − 0.5 and + 0.5 corresponded to mesocephalic type.

### Data collection

The OnDemand 3D software (Cybermed, Seoul, Republic of Korea) was used for morphological measurements of the mandibular angle (height, width, and thickness) and the ITK-SNAP v.3.0 software (Cognitica, Philadelphia, PA) was used to assess the volume of the mandibular angle. Those metrics composed the dependent variables of the study. These assessments were selected by two examiners with at least 2 years of experience in clinical evaluation and diagnosis through CBCT scans, who were previously trained with examples of CBCT scans that were not in the final study sample. The scans were independently evaluated, in a quiet and low-light environment, using the Barco LCD-2124 MDRC monitor (Barco, Kortrijk, Belgium), size 24.1 inches and resolution of 1920 × 1200 pixels. All steps described below were performed for the left and right sides.

#### Width, height, and thickness of the mandibular angle

**Fig. 1:**
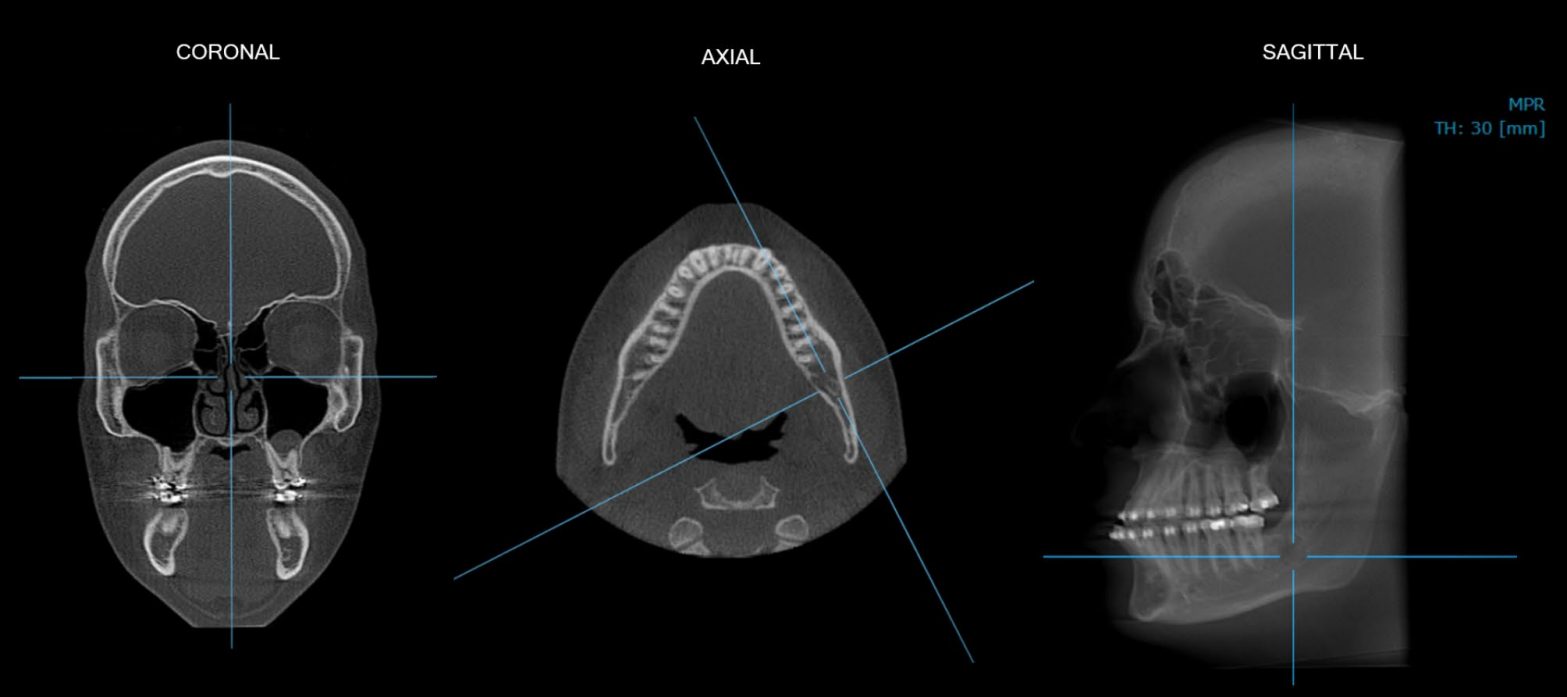
Spatial reorientation of the CBCT multiplanar reconstructions to standardize the assessments of linear measures of the mandible angle

For standardization purposes, prior to the evaluation, each CBCT scan was manually re-oriented as follows: in the coronal view, the vertical reference line of the software was positioned on the median sagittal plane, which is a plane that divides the head into two parts (right and left), passing over the nasal septum. So, in axial reconstruction, the line corresponding to the sagittal plane was aligned with the mandibular body of the side to be analyzed (right or left). Then, in the sagittal reconstruction formed, in which it was possible to visualize the mandible and the angle region, the thickness of the image was increased to 30 millimeters (Fig. 1).

**Fig. 2:**
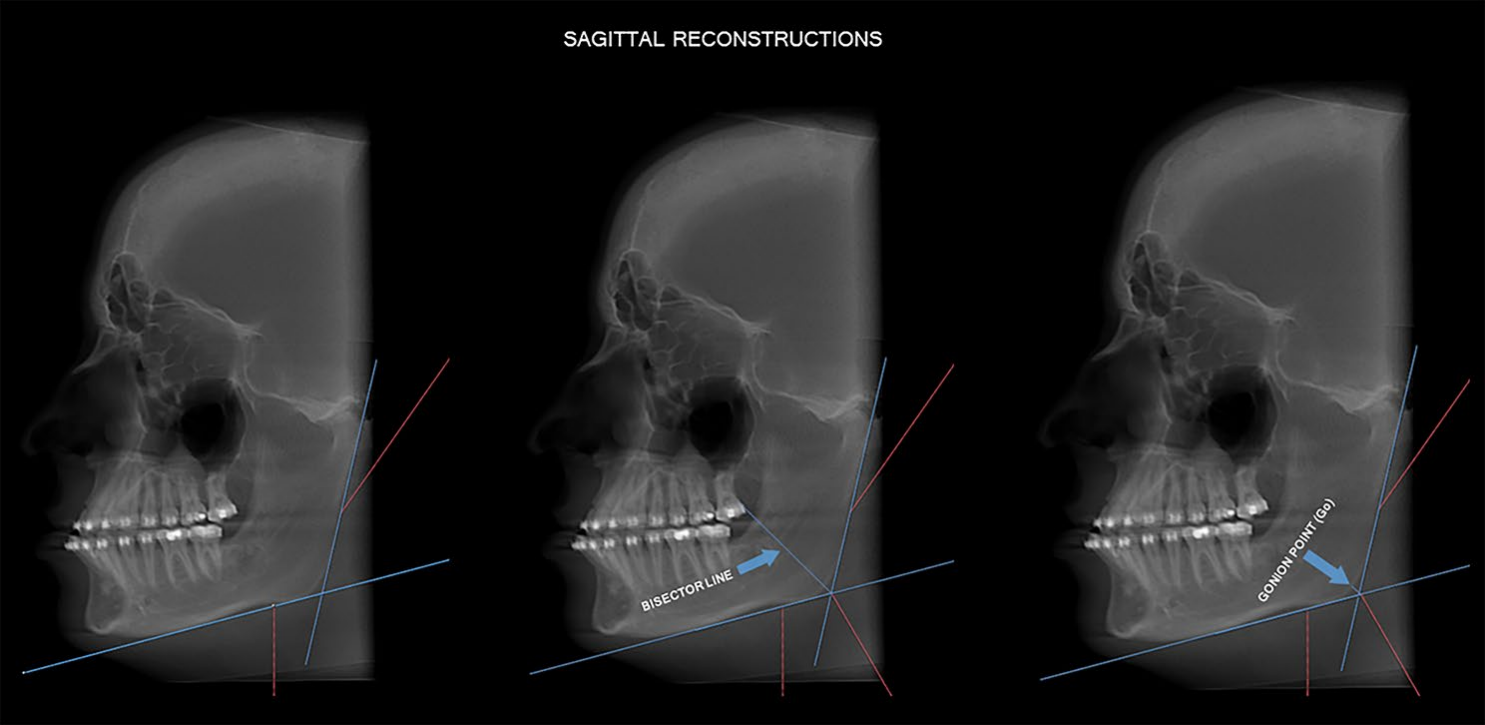
Determination of the Gonion point (Go) to perform the linear measurements (height, width, and thickness) of the mandibular angle

In the parasagittal reconstruction, for demarcation of the Gonion point (Go), two lines were drawn: a vertical line, tangential to the ascending ramus of the mandible and the most posterior point of the mandible head, and a horizontal line, passing through the mandibular bone base. Then, the angle formed by the two drawn lines was determined, with the Go point corresponding to the intersection of the angle bisector with the base of the mandible (Fig. 2).

**Fig. 3:**
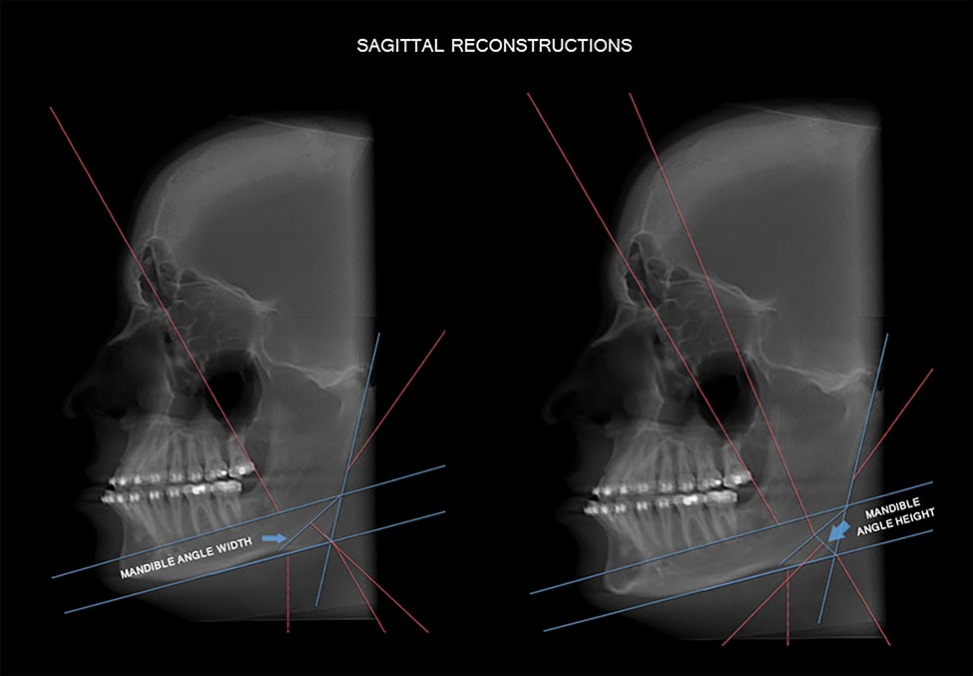
Linear measures of mandibular angle height and width

Subsequently, still in sagittal reconstruction, a horizontal line parallel to the horizontal line of the mandibular bone base was drawn at the uppermost point of the mental foramen. This anatomical landmark was used as a reference for standardizing the volume reorientation and the drafting of the demarcated lines. The width measure of the mandibular angle was defined by a line drawn from the point of greatest concavity of the bone base of the mandible to the point of intersection of the horizontal line of the plane of the mental foramen with the vertical line tangential to the ascending ramus of the mandible. The height measure was obtained from the Go point to the width line of the mandibular angle (Fig. 3).

**Fig. 4:**
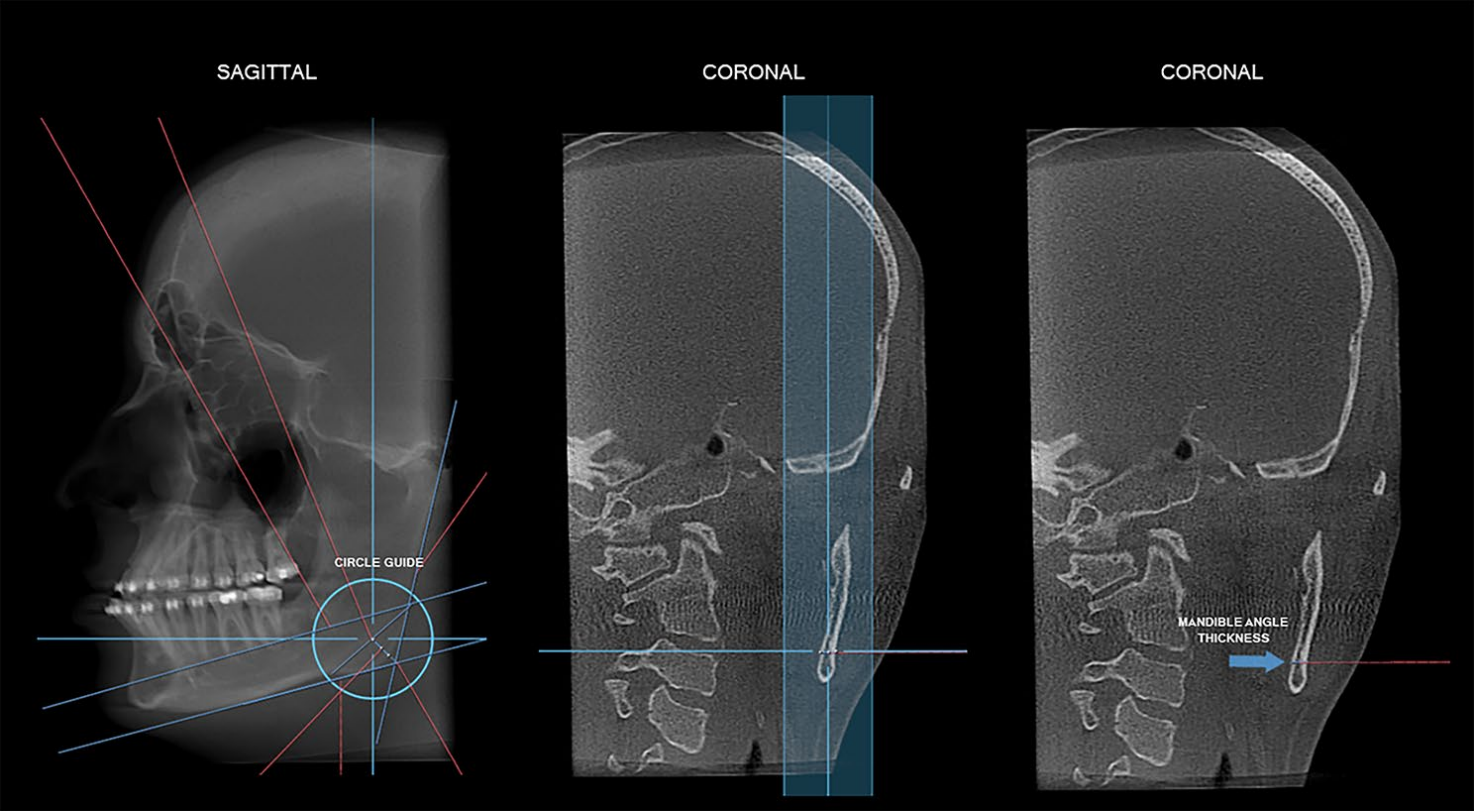
Measurement of mandible thickness

To measure mandibular angle thickness, the line corresponding to the coronal plane, in sagittal reconstruction, was positioned at the uppermost point of the line for determining mandibular angle height. Thus, in coronal reconstruction, a horizontal line was drawn from the external to the internal cortical bone of the mandible (Fig. 4). To measure the thickness of the mandibular angle at the height determined in the previous steps, the “circle guide” tool of the software was used.

#### Volume of the mandible angle

**Fig. 5:**
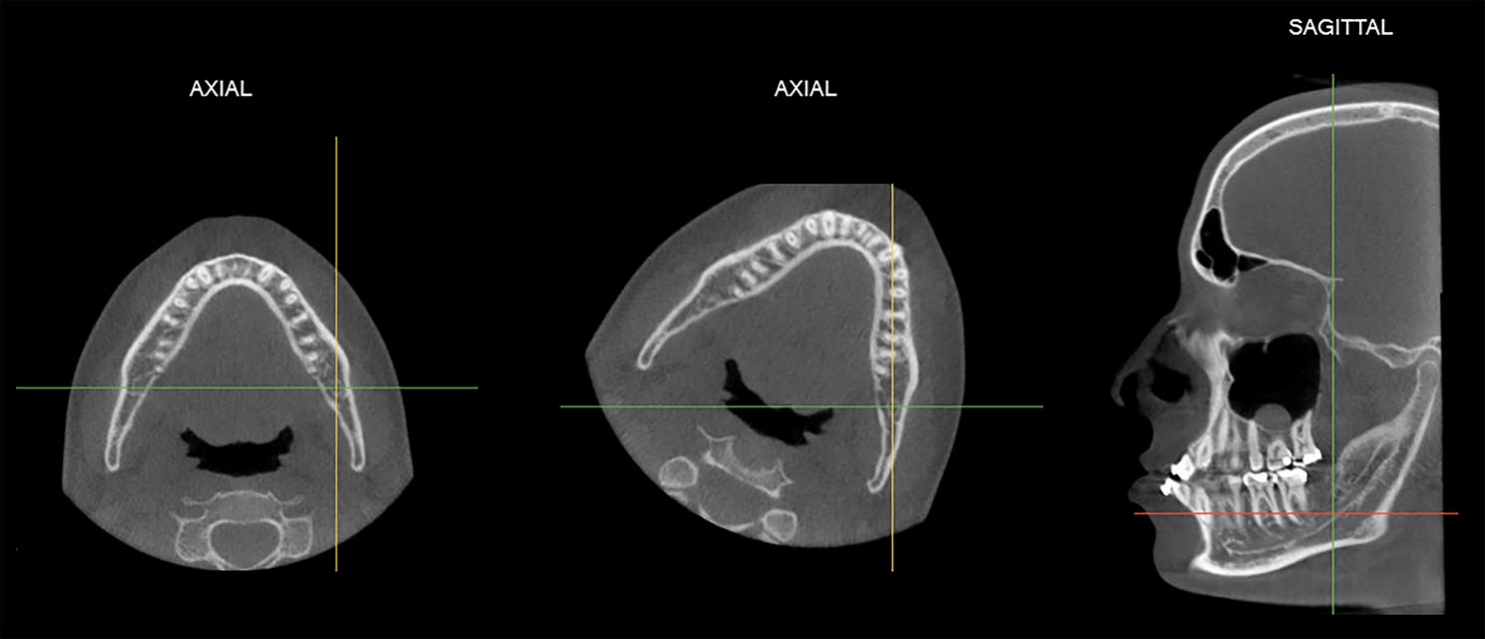
Spatial reorientation of the CBCT multiplanar reconstructions to standardize the assessments of volume of the mandible angle

To standardize the assessments, the CBCT scans were reoriented in the 3D Slicer software (Cambridge, Massachusetts, USA). Initially, in axial reconstruction, the intersection of the vertical and horizontal reference lines were positioned in the region of the mandibular canal on the selected side (right or left). Then, the axial reconstruction was rotated until the vertical reference line crossed the center of the mandibular canal and in the sagittal reconstruction the mental foramen and the mandibular angle were visualized (Fig. 5). Subsequently, the sagittal reconstruction was rotated until the horizontal reference line crossed the upper plane of the mental foramen and the vertical reference line was readjusted until it intersected with the horizontal one in the distal cortical bone of the ascending ramus of the mandible, above the mandibular angle.

**Fig. 6:**
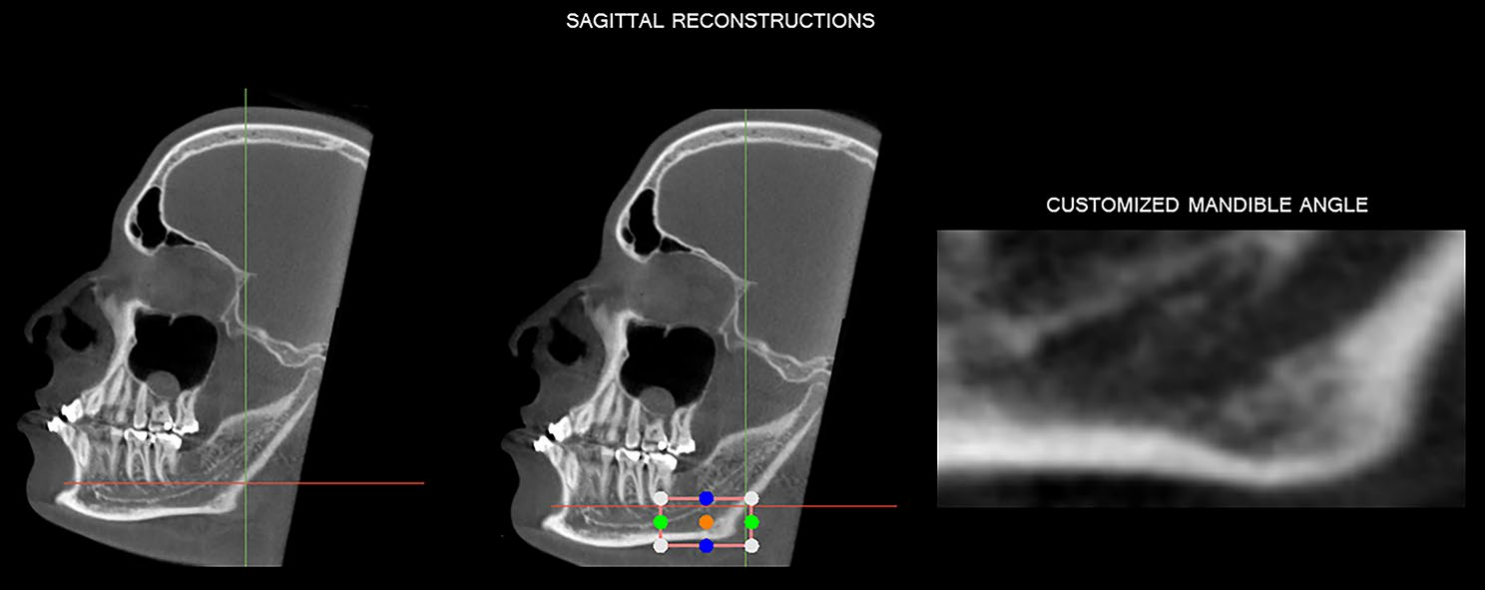
Volumetric rendering to standardize the assessments of volume of the mandible angle

Having the references determined, the “volume redenring” tool was selected in order to individualize the region of the mandibular angle; the tool created a box, allowing the delimitation of the regions of interest, as follows: in the upper region until the reference “blue circle” reached the horizontal line; in the anterior and posterior regions until the “green circle” was positioned in the region of greater curvature in the region of the mandibular bone base and reached the vertical line, respectively, and in the lower region until the reference “blue” circle was in the region of the mandible bone base. Thus, the “crop volume” tool was selected to segment the region of the mandibular angle (Fig. 6).

**Fig. 7:**
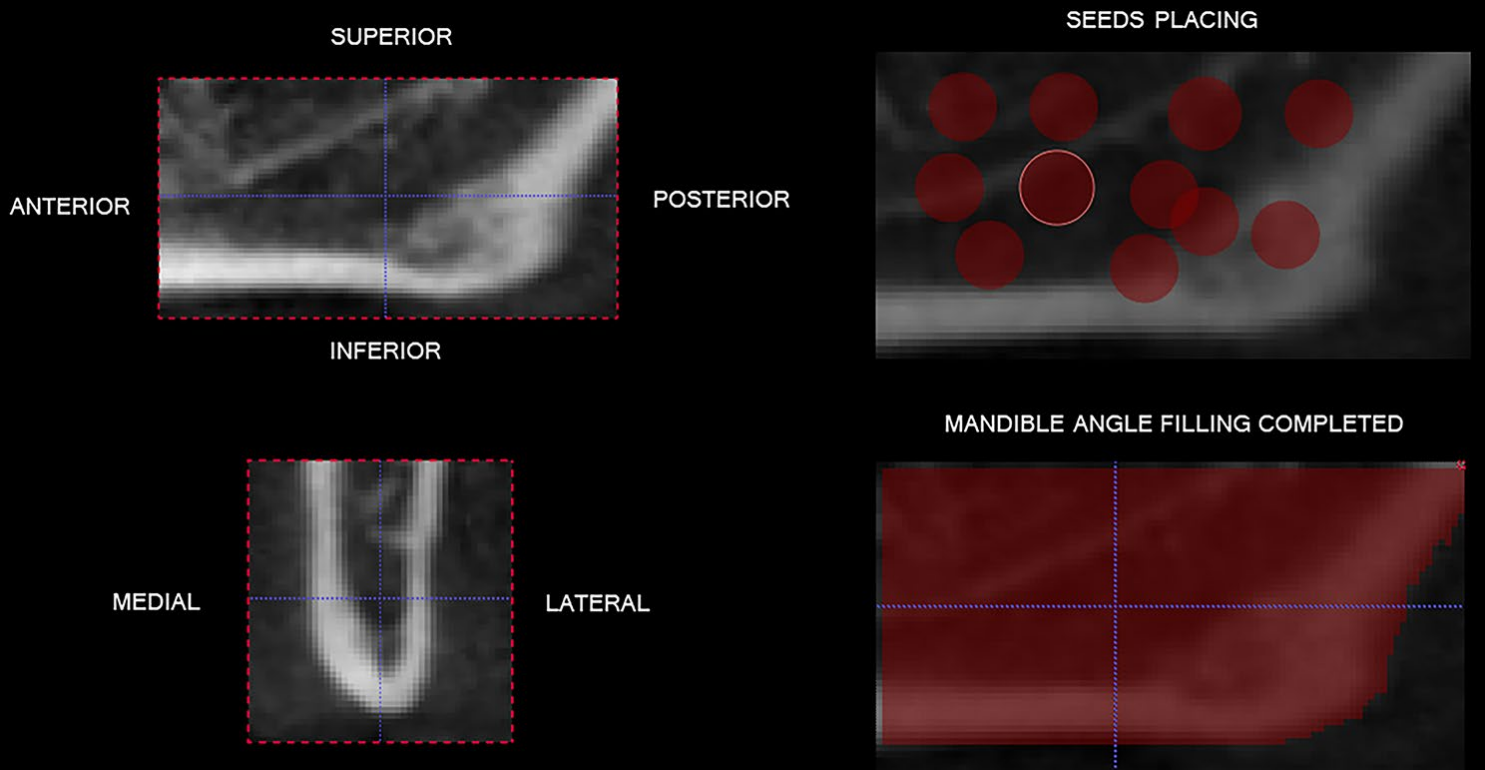
Volumetric evaluation of volume of the mandible angle

Afterwards, the cropped volume was evaluated in the ITKSNAP 3.0 software (Cognitica, Philadelphia, PA), by the semi-automatic segmentation method. After establishing the region of interest (ROI), three interactive and operator-guided steps were performed: first, the “threshold” was established to determine the beginning and end of the segmentation process. The range was − 1805 for the upper threshold and ranged from − 150 to -378 for the lower threshold. After that, “seeds” were placed in the region of interest to start segmentation; and finally, the evolution of segmentation was done by selecting its speed and end. When an area was not well defined, a manual readjustment was performed by the examiner. After the segmentation process, the volume of the structure was provided by the software in mm3 (cubic millimeters) (Fig. 7).

Thirty days after the completion of the assessments, 30% of the sample was reassessed to obtain intra-examiner agreement.

### Data analysis

The intra- and interexaminer agreements of all evaluations (linear measures and volume) were calculated by the Intraclass Correlation Coefficient (ICC) and interpreted according to Koo and Li (2016) [18].

The data of the linear measurements and volume of the mandibular angle were tested for normality by the Shapiro-Wilk test. As they presented normality (p > 0.05), they were compared by Analysis of Variance (multiway ANOVA), with Tukey’s post-hoc test to evaluate the influence of the studied factors (sex, skeletal malocclusion, and facial type). The side was also tested as a factor in the ANOVA; however, since the right and left sides showed no difference between them, they were not treated separately in ANOVA.

For data analysis, the SPSS software version 23.0 (IBM SPSS Statistics for Windows, Version 23.0, IBM Corp, Armonk, NY) was used, considering a significance level of 5%, with test power of 80%.

## Results

After classifying the individuals according to the skeletal pattern of development, the distribution of the sample was as follows: skeletal Class I – n = 126 (62 male and 64 female), skeletal Class II – n = 108 (43 male and 65 female), and skeletal Class III – n = 64 (39 male and 25 female); brachycephalic – n = 122 (63 male and 59 female), mesocephalic – n = 111 (45 male and 66 female), and dolichocephalic – n = 65 (36 male and 29 female). The intra- and interexaminer agreements were good to excellent for linear measures (0.876–0.999) and excellent for volume assessment (0.938–0.958).

Table 1 shows the mean and standard deviation (SD) values of the mandibular angle widths; there was influence of all factors. In regard to sex, male individuals presented higher values than female, regardless of skeletal malocclusion and facial type (p < 0.0001). For skeletal malocclusion, in general, Class III brachycephalic and mesocephalic individuals had higher values in relation to Class I and II skeletal malocclusions (p = 0.003). In relation to facial type factor, class I dolichocephalic female individuals presented higher values than brachycephalic and mesocephalic individuals of the same skeletal malocclusion and sex (p = 0.018).

**Table 1:** Mean values (mm) (SD) mandibular angle width according to sex, skeletal malocclusion, and facial type

Sex	Skeletal malocclusion		Facial type	
		*Brachycephalic*	*Mesocephalic*	*Dolichocephalic*
Female	*Class I*	32.55 (3.13) Bab	32.45 (3.75) Bb	36.5 (2.16) Aa
	*Class II*	31.40 (3.11) b	32.00 (3.24) b	33.28 (2.67) b
	*Class III*	33.68 (3.48) a	35.04 (3.10) a	33.54 (3.43) b
Male*	*Class I*	34.36 (3.82) b	33.73 (5.41)	35.61 (4.71)
	*Class II*	34.18 (4.35) b	34.78 (4.74)	35.04 (4.72)
	*Class III*	37.30 (5.51) a	36.48 (4.79)	35.09 (4.93)

SD: Standard deviation;

* differs from female in all cases

Upper case letters indicate differences between facial types (horizontal) and lower case letters between skeletal malocclusions (vertical)

Table 2 shows the mean and standard deviation (SD) values of the mandibular angle heights; there was also influence of all factors under study. Overall, male individuals had higher height values than female (p < 0.0001). For the skeletal malocclusions, Class III brachycephalic and dolichocephalic male individuals had higher values in relation to Classes I and II (p = 0.021). Regarding the facial type, dolichocephalic individuals presented higher values in relation to brachycephalic and mesocephalic individuals for Class I female and Class III male (p = 0.006).

**Table 2:** Mean values (mm) (SD) mandibular angle height according to sex, skeletal malocclusion, and facial type

Sex	Skeletal malocclusion		Facial type	
		*Brachycephalic*	*Mesocephalic*	*Dolichocephalic*
Female	*Class I*	5.42 (1.69) B	6.03 (1.56) AB	7.13 (1.99) A
	*Class II*	6.20 (1.77)	6.10 (1.32)	6.35 (1.74)
	*Class III*	5.69 (1.85)	6.58 (1.18)	6.26 (1.61)
Male	*Class I*	6.49 (1.57) b*	6.23 (1.69)	7.34 (1.85) b
	*Class II*	7.48 (2.36) ab*	6.85 (1.67)*	7.77 (2.51) b*
	*Class III*	7.76 (1.97) ABa*	6.72 (1.52) B	9.22 (3.14) Aa*

SD: Standard deviation;

* differs from female within the same skeletal malocclusion and facial type;

Upper case letters indicate differences between facial types (horizontal) and lower case letters between skeletal malocclusions (vertical)

Table 3 shows the mean and standard deviation (SD) values of the mandibular angle thicknesses. There was significant influence only of the sex, in which male individuals presented higher values for mandibular angle thickness than female for Class I dolichocephalic and Class III mesocephalic individuals (p = 0.005). There was no influence of skeletal malocclusion (p = 0.192) and facial type (p = 0.341).

**Table 3:** Mean values (mm) (SD) mandibular angle thickness according to sex, skeletal malocclusion, and facial type

Sex	Skeletal malocclusion			Facial type	
		*Brachycephalic*		*Mesocephalic*	*Dolichocephalic*
Female	*Class I*	5.53 (0.77)		5.42 (1.16)	5.43 (1.04)
	*Class II*	5.64 (1.20)		5.16 (1.23)	5.64 (0.93)
	*Class III*	5.64 (1.06)		4.60 (0.88)	5.54 (1.76)
Male	*Class I*	5.66 (1.08)		5.76 (1.10)	6.15 (0.91)*
	*Class II*	5.23 (1.05)		5.69 (1.31)	5.94 (1.26)
	*Class III*	5.42 (1.16)		6.03 (1.44)*	5.50 (0.95)

SD: Standard deviation;

* differs from female within the same skeletal malocclusion and facial type

Table 4 shows the mean and standard deviation (SD) values of the volume of the mandibular angle. Among the factors studied, there was influence only of sex (p < 0.0001), in which male individuals presented higher volumes than female regardless of skeletal malocclusion and facial type. There was no influence of skeletal malocclusion (p = 0.467) and facial type (p = 0.900).

**Table 4:** – Mean values (mm³) (SD) mandibular angle volume according to sex, skeletal malocclusion, and facial type

Sex	Skeletal malocclusion			Facial type	
		*Brachycephalic*		*Mesocephalic*	*Dolichocephalic*
Female	*Class I*	1.59 (0.37)		1.52 (0.48)	1.49 (0.37)
	*Class II*	1.72 (0.61)		1.51 (0.43)	1.54 (0.44)
	*Class III*	1.50 (0.38)		1.45 (0.53)	1.46 (0.37)
Male*	*Class I*	1.91 (0.57)		2.04 (0.84)	2.12 (0.63)
	*Class II*	2.16 (0.63)		1.99 (0.52)	2.20 (0.66)
	*Class III*	2.15 (0.83)		2.46 (0.97)	2.03 (0.82)

SD: Standard deviation;

* differs from female in all cases

## Discussion

The development of the mandibular angle is influenced by the action of biomechanical factors acting on the morphology of the mandible, which directly influences its size. Considering that the angle of the mandible is a structure of the craniofacial complex influenced by the masticatory muscles and mandible elevators, the authors hypothesized that this anatomical region could be affected by variations in craniofacial development. There was a significant influence of the sex on all variables analyzed (height, width, thickness, and volume of the mandibular angle), in which in general male individuals presented higher values than female. The skeletal malocclusion and facial type factors influenced only the width and height variables; in general, the skeletal malocclusion Class III and dolichocephalic individuals presented higher values than the other types of malocclusions and facial types.

Previous studies that assessed the influence of sex on mandibular structures, such as mandibular head and mandibular angle, or other structures of the craniomaxillofacial complex, also found that measurements in males are greater than in females, corroborating our results [1, 4, 8, 9, 12, 19, 20]. It is believed that the difference between male and females individuals is due to the fact that men tend to have larger and denser bones than women, because they undergo greater bone remodeling mainly due to masticatory forces [8, 19]. Another important factor to mention is age, but previous studies have found no relationship between this factor and mandibular angle [1, 12, 19, 21]. Despite the existing theory on bone remodeling with the aging of the individual, what is still known is that not much is altered in the mandibular angle region in relation to the mandibular body [1, 21]. Even in cases of edentulous patients without associated bone disease, the mandibular angle region was not changed significantly with age [1, 22]. These findings caused the authors not to include age as a factor to be studied.

Regarding the skeletal malocclusion factor (Class I, II, and III), the significant association was found only for the width and height variables, in which Class III individuals presented higher values in relation to Classes I and II types in some subgroups. Despite the different methodologies adopted, the studies of Arieta-Miranda et al. (2013), [23] and Miranda-Viana et al. (2021) [8] found that discrepancies and imbalances in craniofacial development and growth of individuals can lead to morphological changes in the craniomaxillofacial complex, such as the region of the hard palate and mandibular head. Thus, it seems that the more anterior positioning of the mandible in Class III individuals can promote higher values in linear measures of height and width of the mandibular angle. According to the literature reviewed, this is the first study to assess the relation between mandibular angle height and width and skeletal malocclusion in CBCT scans. Thus, it is difficult to compare our results directly with previous studies.

Facial type also influenced the width and height variables, and dolichocephalic individuals had higher values in relation to brachycephalic and mesocephalic types in some cases. This can be explained by the imbalance in the vertical trend of growth and development of the craniomaxillofacial complex, consistently with the results of previous studies [8, 9]. On the other hand, the studies of Gomes et al. (2015) [19] and Lemes et al. (2021) [24] found no association between the different facial types in the measures of width/height of the coronoid process and ascending ramus of the mandible, respectively. However, it a direct comparison is not possible, since the structures evaluated and the methodologies adopted were different, in addition to discrepant sample sizes of the above studies (n = 132 and n = 159, respectively). In this study, the mandibular angle was assessed in 298 patients, proportionally distributed between the different sexes and skeletal patterns of development, based on CBCT scans that provide 1:1 image, with no magnification or distortion. For the measurements, the reference was the gonion point, which marks the region of the mandibular angle, and according to Mendoza et al. (2018), [7] using this point enables more reliable comparisons between the studies that use this same criterion.

Regarding the investigated variables of thickness and volume of the mandibular angle, there was no influence of the different skeletal patterns of development (skeletal malocclusions and facial types), which corroborates previous studies found in the literature [25, 26]. In contrast, the study conducted by Olbrisch et al. (2022) [27] found significant association between the different skeletal patterns of development and mandibular volume. In addition to the different methodologies applied and smaller sample size (n = 111) in relation to the present study, the authors assessed the volume of the mandibular body; thus, it is not possible to directly compare the results.

Although we did not assess the skeletal asymmetry of the individuals in this study, this factor may be related to our findings regarding the mandibular angle. Mandibular asymmetry is characterized by dimensional differences in size, shape, and volume of the left and right sides of the mandible, which can be the cause of aesthetic and functional problems. The imbalance in the process of craniofacial development and growth of the individual may also be associated with skeletal asymmetry [27]. Previous studies have observed that individuals with different skeletal development patterns, such as Class III malocclusion and dolichocephalic, presented higher values of skeletal asymmetry [7, 12]. Future longitudinal studies investigating this clinical relationship are encouraged.

Because this is a cross-sectional study, it was only possible to assess the influence of the factors studied, but not to estimate causal relations. Also, since it is a study based on a convenience sample and CBCT scans, the aesthetic and functional characteristics of the patients were unable to be directly correlated. However, the literature shows correlation of masticatory force and/or muscle volume with changes in bone width and height [28–30]. This information corroborates the results of the present study. Given the imbalance in the growth process and craniofacial development, the muscle and bone tissues attempt to compensate for this imbalance. Despite this limitation and considering the results, it is important to note that the sample size of this study was robust, and homogeneously distributed within each variable evaluated, which provided reliable results. As well, the excellent intra- and interexaminer agreements are also highlighted. Thus, it can be considered that possible variations of the evaluators did not affect the results and conclusions of the study.

The results of the present study may help the professionals to identify how the skeletal pattern of development and/or sex can influence the shape/volume of the mandibular angle region. The surgeons may ponder if a different/special corrective approach is necessary. Knowledge about imbalance in the trend of craniofacial growth and development, the different skeletal patterns, and their correlation with the measurements of mandible angle width and height, can provide viable information for orthodontic and surgical interventions associated with this anatomical region.

## Conclusion

The variations in the craniofacial growth pattern, considering the different skeletal malocclusions and facial types, had some influence in the width and height dimensions of the mandibular angle. In addition, the sex influenced all the mandibular angle variables studied (width, height, thickness, and volume), with high values for males. Thus, professionals should be aware of possible changes in the height and width of the mandibular angle depending on the patient’s developmental characteristics. Also, may ponder if a different/special corrective approach is necessary.

## Data Availability

The datasets used and/or analyzed during the current study are available from the corresponding author on reasonable request.
